# High-Density Nanowells Formation in Ultrafast Laser-Irradiated Thin Film Metallic Glass

**DOI:** 10.1007/s40820-022-00850-4

**Published:** 2022-04-13

**Authors:** Mathilde Prudent, Djafar Iabbaden, Florent Bourquard, Stéphanie Reynaud, Yaya Lefkir, Alejandro Borroto, Jean-François Pierson, Florence Garrelie, Jean-Philippe Colombier

**Affiliations:** 1 Univ Lyon, UJM-Saint-Etienne, CNRS, Institute of Optics Graduate School, Laboratoire Hubert Curien, UMR CNRS 5516, 42023 St-Etienne, France; 2 Université de Lorraine, CNRS, IJL, 54000 Nancy, France

**Keywords:** Nanowell, Nanoripple, Metallic glass, Surface functionalization, Femtosecond laser

## Abstract

We present an effective approach for fabricating nanowell arrays in a one-step laser process with promising applications for the storage and detection of chemical or biological elements. Biocompatible thin films of metallic glasses are manufactured with a selected composition of Zr_65_Cu_35_, known to exhibit remarkable mechanical properties and glass forming ability. Dense nanowell arrays spontaneously form in the ultrafast laser irradiation spot with dimensions down to 20 nm. The flared shape observed by transmission electron microscopy is ideal to ensure chemical or biological material immobilization into the nanowells. This also indicates that the localization of the cavitation-induced nanopores can be tuned by the density and size of the initial nanometric interstice from the columnar structure of films deposited by magnetron sputtering. In addition to the topographic functionalization, the laser-irradiated amorphous material exhibits structural changes analyzed by spectroscopic techniques at the nanoscale such as energy-dispersive X-ray spectroscopy and electron energy loss spectroscopy. Results reveal structural changes consisting of nanocrystals of monoclinic zirconia that grow within the amorphous matrix. The mechanism is driven by local oxidation process catalyzed by extreme temperature and pressure conditions estimated by an atomistic simulation of the laser-induced nanowell formation.

## Highlights

- Ultrafast laser-induced nano-topography modifications: generation of highly concentrated 20 nm diameter nanowells on the surface with expected applications for storage of chemical and biological active species and for blocking crack propagation.
- Ultrafast laser-induced structural modifications: turning of a metallic glass to a composite material of monoclinic zirconia crystallites embedded inside amorphous metallic glass.
- A flexible one-step laser irradiation process without direct mechanical contact for thin film metallic glasses surface functionalization.

## Graphical abstract

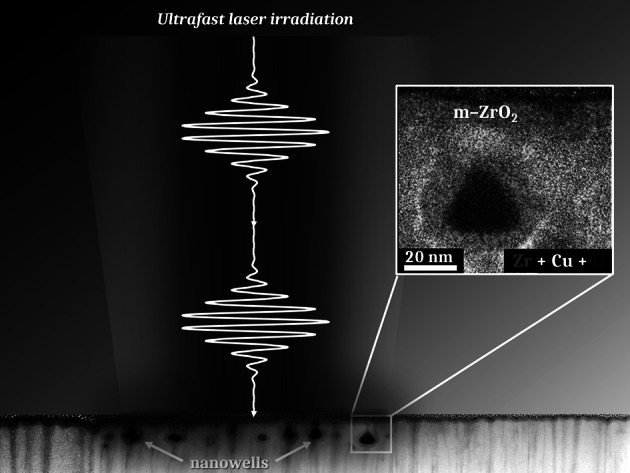

## Introduction

The miniaturization of surface structures is a perpetual challenge as it expands the possible applications in nanotechnologies. From quantum dots to arrays at the nanometer scale, innovative methods for creating miniaturized structures (micronic and submicronic) have been developed [1], namely lithography or photolithography techniques, self-assembly, laser etching or even anodization process and gas exposure [2–7]. However, for all applications domains, the size, number and distribution of the generated nanostructures must be controlled. Nanowells (NWs), consisting of high aspect-ratio surface pores at the nanoscale, represent an ideal type of structuring for many applicative fields and for a wide range of materials, from polymers [8] to metallic and composite materials [9]. Arrays of nanowells have been successfully used to store elements [7, 10], to modulate the optical surface properties of the material [9, 11] or even for the detection of biological and chemical components [12–15]. The sensing function represents one of the most attractive properties of NWs arrays since their distribution and small size offer a possible reduction in the chemical reagent quantities used and thus a general decrease in cost and generated waste [4]. Therefore, a fast and reproducible process for obtaining this type of array is a major challenge for many applications in various fields, such as biochemistry, biomedicine, and renewable energies.

Laser texturing is a well-known "one-step" process for the surface functionalization of any type of material in a controlled and reproducible way. In addition to the well-established applications of laser-induced periodic surface structures (LIPSS) [16, 17], novel singular and smaller scale topographies such as nanopeaks or nanocavities have recently been reported upon ultrafast laser-metal interaction [18, 19]. Irregularly distributed nanocavities are shown to appear due to subsurface cavitation phenomena, but this mechanism driven by field-enhancement on random roughness cannot be readily controllable [20–24]. Very specific irradiation conditions are required in order to favor the appearance of organized nanostructures resulting from hydrodynamic instabilities, but their emergence on the pristine irradiated surface requires a quasi-irreproachable material surface state, restricting the material possibly functionalized [17].

Metallic glasses (MGs), or amorphous metals, are known for their particularly smooth surface quality due to their lack of classical large-scale crystal defects such as dislocations or grain boundaries. In that respect, the irradiation of bulk metallic glasses allows for the generation of classical periodic surface structures that are regular and show few bifurcations [25–28]. In addition to their surface condition limiting undesired scattering effects, amorphous metals combine the properties of both crystalline metals and glassy materials [29, 30]. Consequently, they have already been implemented in many advanced applications such as micromachining, microelectronics [31] or in the biomedical field with surgical tools or implants [31, 32]. Thin film metallic glasses (TFMGs) broaden the applicative possibilities of these materials, in particular with a wider range of compositions from binary systems to more complex 5-element systems as for their bulk counterparts [33, 34].

In this work, thin film metallic glasses are fabricated by magnetron sputtering and then irradiated by ultrafast laser pulse to generate an array of nanowells with a highly uniform concentration of 600 µm^−2^ and an average size of 30 nm in depth and 20 nm in diameter. Well below the optical diffraction limit, inhomogeneous laser coupling to the surface spontaneously triggers the arrangement of hundreds of wells at the nanoscale within the laser spot. A Zr-Cu metallic glass system is investigated here owing to its valuable mechanical and recognized biocompatible properties and its good glass forming ability [35]. The specific columnar morphology of these thin films is controllable through the deposition parameters and compositions used [36] and has an important impact on the development of nanowells after laser irradiation. The whole process mastering, from the fabrication of the TFMGs to their irradiation, allows us to obtain the desired NWs features. The developed process suggests that the size, concentration and distribution could be tuned for dedicated functions. The resulting surface structures are analyzed by scanning electron microscopy (SEM) and scanning transmission electron microscopy (STEM) coupled with elemental analyses such as energy-dispersive X-ray spectroscopy (EDS) and electron energy loss spectroscopy (EELS) to observe the structural modifications. Image analysis is also carried out in order to clarify the characteristics of the nanowells created and to detect the crystalline structures generated after laser irradiation. A functionalization of the thin film metallic glasses by generating NWs on their surface would be an additional major asset for many applications in the nanotechnology field.

## Initial Material Surface Features and Laser Irradiation Strategy

**Fig. 1:**
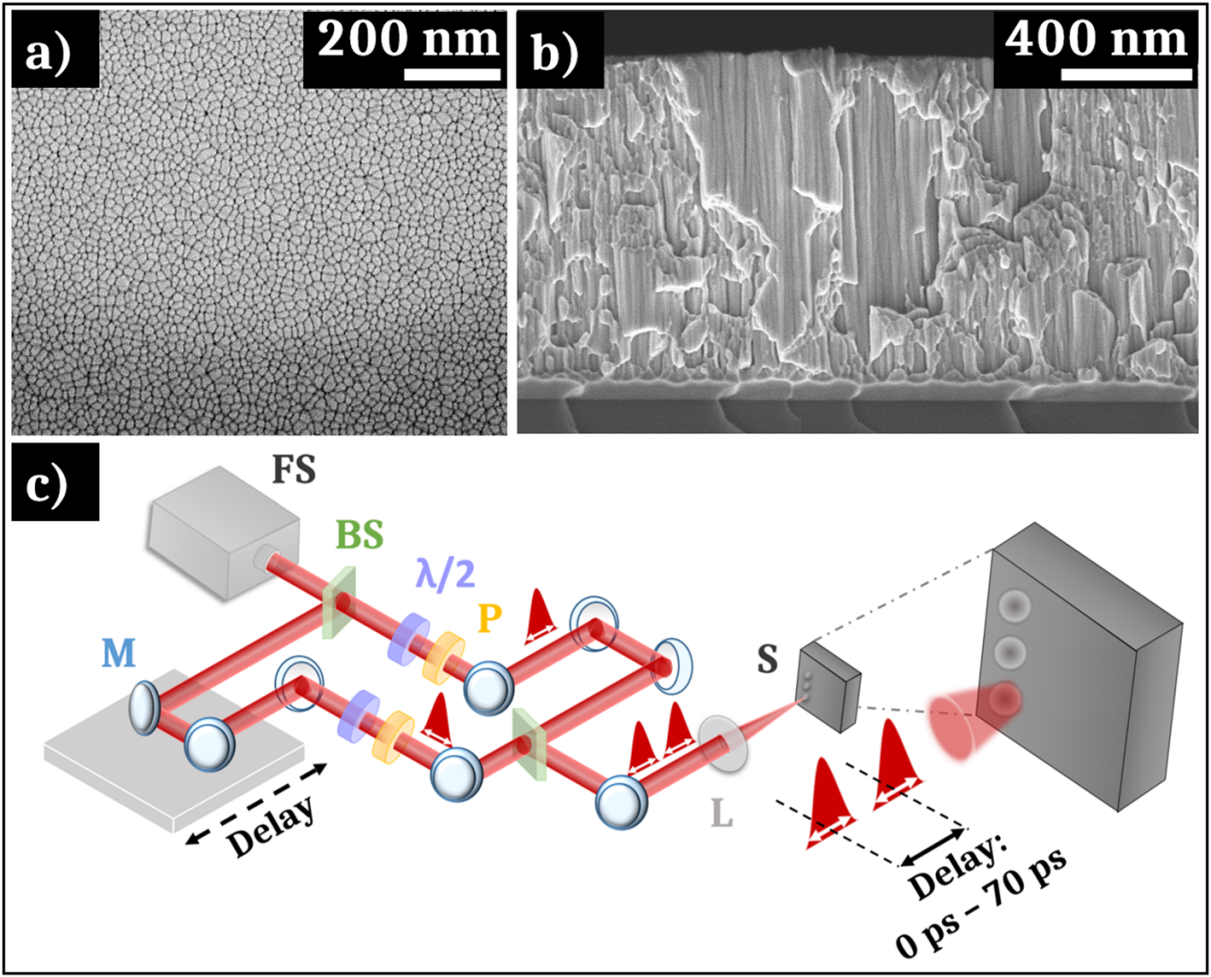
SEM image of **a** the surface and **b** the cross section of the non-irradiated Zr-Cu thin film metallic glass manufactured by magnetron sputtering. **c** Drawing of the experimental setup used to generate single or double femtosecond pulses with controlled delay and polarizations (*FS* represents the femtosecond laser system, *BS* a beam splitter, *λ*/2 a have-wave plate, *P* a polarizer, *M* a mirror, *L* a 250 mm lens and *S* the sample)

In this study, the manufactured sample consists of a thin film metallic glass presenting a Zr_65_Cu_35_ composition given in nominal atomic percent. The film is obtained by magnetron co-sputtering of zirconium and copper targets under an argon atmosphere. The working pressure is 1 Pa and the targets to substrate distance are fixed at 9 cm. The composition of the film is adjusted by controlling the discharge current applied to the targets. The substrate, a (100) silicon wafer, is in rotation in order to homogenize the element concentration. The film is deposited without external heating. At the end of the process, the obtained thin film metallic glass is characterized by XRD in order to check its amorphous structure. As expected, the composition fits within the reported range of stable amorphous compositions obtained by magnetron sputtering [36]. Figure 1a–b presents, respectively, a SEM image of the surface morphology and of a cross section of the sample. The observed columnar morphology is typical of a sample obtained by sputtering in these elaboration conditions [36]. This morphology, namely the width of the columns and surface smoothness, can be modified by tuning the argon working pressure and the reduced temperature $T_{r} = T/T_{m}$ (with *T* the deposition temperature and *T*_*m*_ the melting point of the alloy).

The ultrashort laser experiments are performed in air using a Ti:sapphire femtosecond laser, generating linearly polarized pulses with a central wavelength *λ* = 800 nm. The pulse duration is fixed at 60 fs. Using the *D*^2^ technique [37], the single shot damage threshold is evaluated at 0.08 J cm^−2^ with a spot size of the Gaussian profile at (1/e^2^) of 2*ω*_0_ = 62 µm (where *ω*_0_ is the beam waist). Figure 1c shows the schematic diagram of the experiment. Double pulses, with controlled energy and polarization in each optical path, can be generated using a Mach–Zehnder interferometer configuration. In one optical path, a delay line generates a temporal separation Δ*t* between the two pulses up to 70 ps. The sample is placed at the focal plane of a 250 mm lens. For the single pulse experiments, one optical path is blocked and different conditions of fluence and pulse number are tested.

The SEM micrographs of the different laser-irradiated areas are analyzed by an image analysis method using the OpenCV library. Firstly, a thresholding is carried out on the images to obtain a binary image. Then, a method of detection and differentiation of objects based on union-find algorithms is used to detect the NWs [38].

To understand the thermomechanical effects induced by ultrafast laser irradiation on NWs formation dynamics and give a clear picture about the transition from in-depth trapped voids to flared holes, a molecular dynamics (MD) investigation is performed neglecting any chemical activity. Femtosecond laser interaction with the metallic glass surface is modeled using the two-temperature model (TTM) implemented in the LAMMPS software [39] via the USER-MISC package [40]. Heat transfer through and between electronic and atomic subsystems at the picosecond timescale is then coupled to classical force field acting between atoms of the amorphous material. For this purpose, an initial MG simulation box (813.83 × 14.28 × 12.40 nm) with the composition Zr_66.7_Cu_33.3_ is prepared with a free boundary condition following the longitudinal laser direction *x* and periodic boundary conditions on the transverse directions *y* and *z*. This calculation box is divided into two distinct 400 nm regions along *x*, the vacuum and the MG thickness wherein the 100 nm rear side serves as absorbing/non-reflecting boundary conditions. To mimic the presence of interstices between the TFMG columns along the *x*-direction, a 1 nm diameter cylinder is inserted. Before depositing the electromagnetic energy on the surface, the MG structures are relaxed under isothermal-isobaric ensemble (NPT) at 300 K and 1 bar pressure for 10 ps. Then the TTM-MD stage [41, 42] takes place during 15 ps with a time step of Δ*t* = 1 fs. Finally, the system is evaluated by MD for an extra 150 ps duration using micro-canonical ensemble (NVE). To observe the voids formation, the laser conditions are fixed as close as possible to the experimental data. Preliminary ellipsometry measurements gave the complex optical index for cold material of $\tilde{n} = n + ik = 2.6 + 3i$. For the simulation, the skin depth is fixed to 15 nm considering the expected transient change of $\tilde{n}$ due to the induced electron–phonon nonequilibrium during the laser pulse deposition. The absorbed fluence is fixed to 60 mJ cm^−2^ and a 60 fs-pulse duration is used. The embedded atom model (EAM) potential employed was developed by M.I. Mendelev et al. [43] and the thermodynamic quantities mapping are obtained using the OVITO software [44].

## Topographical Structural and Chemical Features of Nanowells

**Fig. 2:**
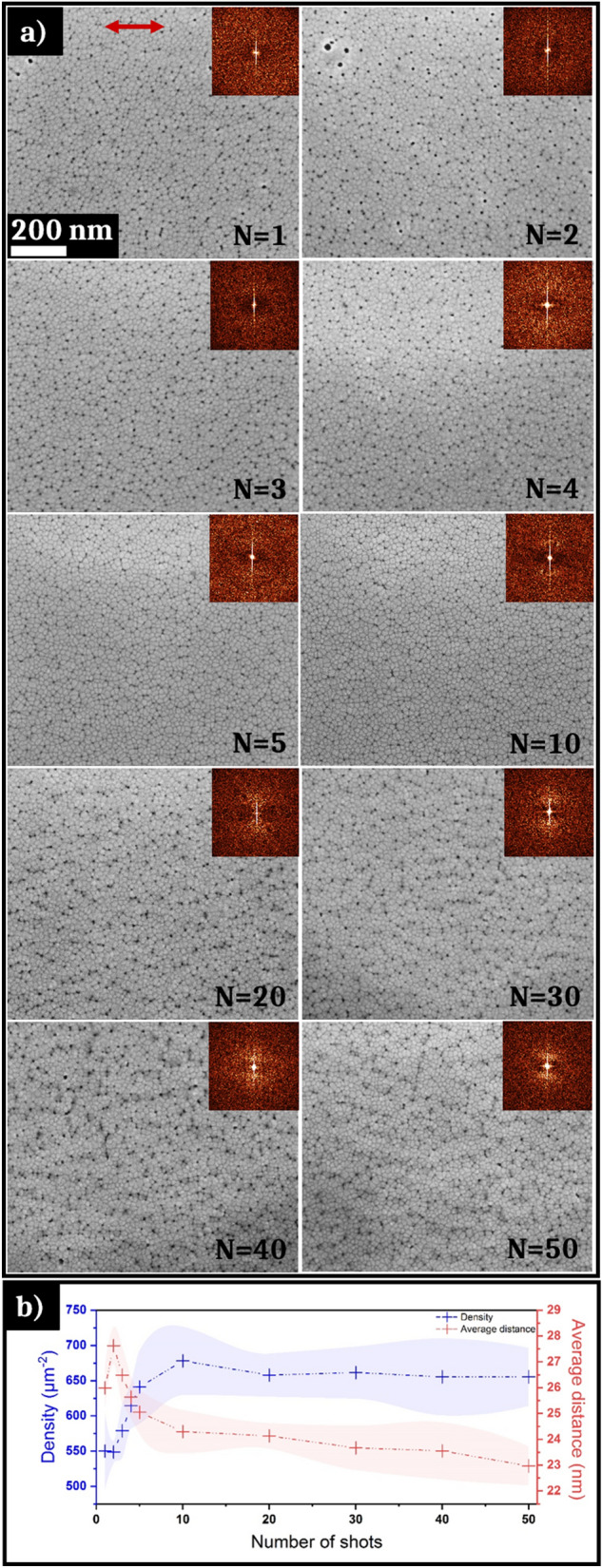
**a** SEM images of the nanowells microstructure obtained in the femtosecond laser area irradiated under the fluence of 0.06 J cm^−2^ for different numbers of laser shots (1, 2, 3, 4, 5, 10, 20, 30, 40, and 50) with 2D-Fourier transforms as insets; the red arrow represents the polarization of the electric field. **b** Evolution of the density of nanowells and of the average distance between the nanowells as the number of laser shots increases. The measurement dispersion is represented by a shaded area around the values

Before laser irradiation, TFMGs present very low average RMS (root-mean-square) roughness, less than 2 nm, measured by atomic force microscopy (AFM). High roughness promotes the appearance of large periodic structures with many irregularities and bifurcations. Working with a very smooth surface state with low roughness is thus suitable for creating self-organized multi-scale structures under optimal conditions [45]. The fluence conditions used for this work are below the single shot damage threshold found for the TFMG, from 0.04 to 0.07 J cm^−2^. Figure 2a presents SEM pictures of the evolution of the surface microstructure of the TFMG after different number of laser shots at a 0.06 J cm^−2^ fluence. While the initial surface morphology seems to be globally unaltered after irradiation, having the same columnar shape, dark dots that will be identified as nanowells appear between the interstices, as can be seen in Fig. 2. The nanowells density changes non-monotonously with the number of pulses, as shown on the graph in Fig. 2b displaying the evolution of the amount of nanowells for different numbers of laser pulses and the evolution of the average distance between the nanowells. Starting from a density of 550 nanowells per µm^2^ for 1 shot, the density increases until 680 µm^−2^ between 2 and 10 laser shots. Beyond this threshold, a stabilization of the density of NWs around 650 µm^−2^ is visible until 50 shots. In parallel with the evolution of the nanowells concentration, the distance between them varies between 20 and 30 nm. As expected, the evolution of the average distance between NWs follows the inverse of their density and, as the concentration increases, the distance decreases and vice versa.

The 2D-Fourier transforms of the SEM images depicted as insets on Fig. 2a show an absence of any spatial order from 1 to 5 laser shots. From 10 shots up to 50 shots, a preferential horizontal orientation is observable with the presence of two vertical bright dots. This order appears parallel to the polarization of the electric field and intensifies with the number of laser shots. The period of these structures is close to 100 nm, and regarding their orientation, these nanoripples coincide with the first stage of usually reported high spatial frequency LIPSS formation [46, 47].

**Fig. 3:**
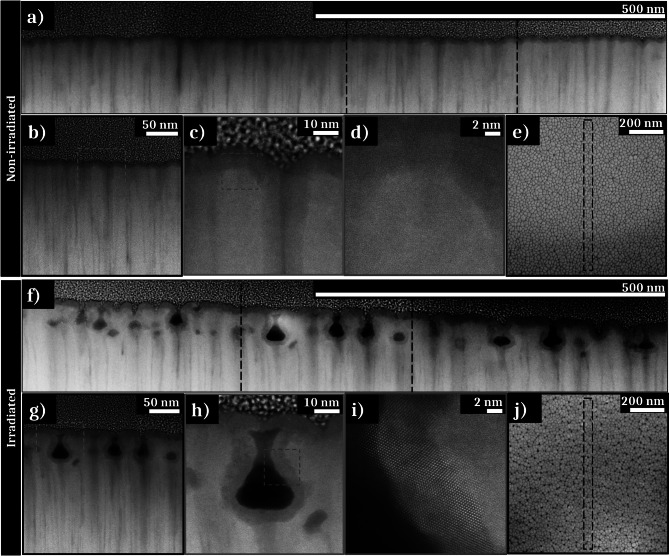
**a**–**d** High-Angle Annular Dark-Field (HAADF) STEM images of a FIB lamella extracted from a non-irradiated area showing the initial columnar morphology of the thin film; **d** the lack of visible order is characteristic to an amorphous structure; **e** SEM picture of the FIB lamella extracted zone from the non-irradiated sample. **f**–**i** HAADF STEM images of a FIB lamella extracted from a textured area irradiated under the fluence of 0.06 J cm^−2^ with 50 pulses, showing nanowells on the sub-surface of the thin film. **h** shows an “open” nanowell. **i** shows columns of atoms characteristic of a crystalline structure surrounding this nanowell. **j** SEM picture of the FIB lamella extracted zone from the irradiated sample

In order to fully characterize these nanowells, two FIB lamellas are extracted from a non-irradiated zone (Fig. 3e) and from an irradiated zone exposed to a fluence of 0.06 J cm^−2^ with 50 pulses (Fig. 3j).

Different zooms of the FIB lamella extracted from the non-irradiated zone are presented in Fig. 3a–d with high-angle annular dark-field (HAADF) STEM images. The lower part of the images displays the sample, on which is deposited a platinum layer and a carbon layer, necessary to realize the FIB lamella extraction. A dark zone is noticeable in the 10 first nanometers under the surface sample. The difference in contrast visible as vertical lines (Fig. 3b) is due to a change of density of elements, of structure, or the presence of some defects, i.e., the column boundaries. The dark lines could also appear during the FIB lamella extraction (curtain effect). By comparison between Fig. 1b and 3a, it is established that the contrasting lines visible in the STEM image correspond to the interstices between the columns formed during deposition. This initial columnar morphology is clearly visible on the surface of the sample on the STEM picture, in accordance with the very low initial surface roughness measured by AFM. The zoomed picture exposed on Fig. 3d exhibits an amorphous structure characterized by an absence of atomic order.

The second FIB lamella extracted from the irradiated zone presents NWs, regularly disseminated all over the lamella (Fig. 3f). The NWs are located close to the sample surface (~ 50 nm from the surface). Some of them seem to be attached to the surface (“open” NWs), whereas others appear to be detached (“closed” NWs). In fact, these “closed” nanowells represent either nanowells where the chimney is not visible (because of the location of the FIB extraction), or cavitation bubbles in the subsurface that have not burst to form “open” nanowells. The NWs exhibit a diameter between 10 and 30 nm. As seen for the non-irradiated zone, some contrast variations are observable in the STEM pictures on the sample surface and around the nanowell. Moreover, in this case, the high-resolution STEM (HR-STEM) image (Fig. 3i) features atomic lattices around the nanowells. This is characteristic of a crystalline structure surrounding the wells and on the surface of the irradiated area. Remarkably, the NWs appear to be preferentially formed in boundary regions between columns.

**Fig. 4:**
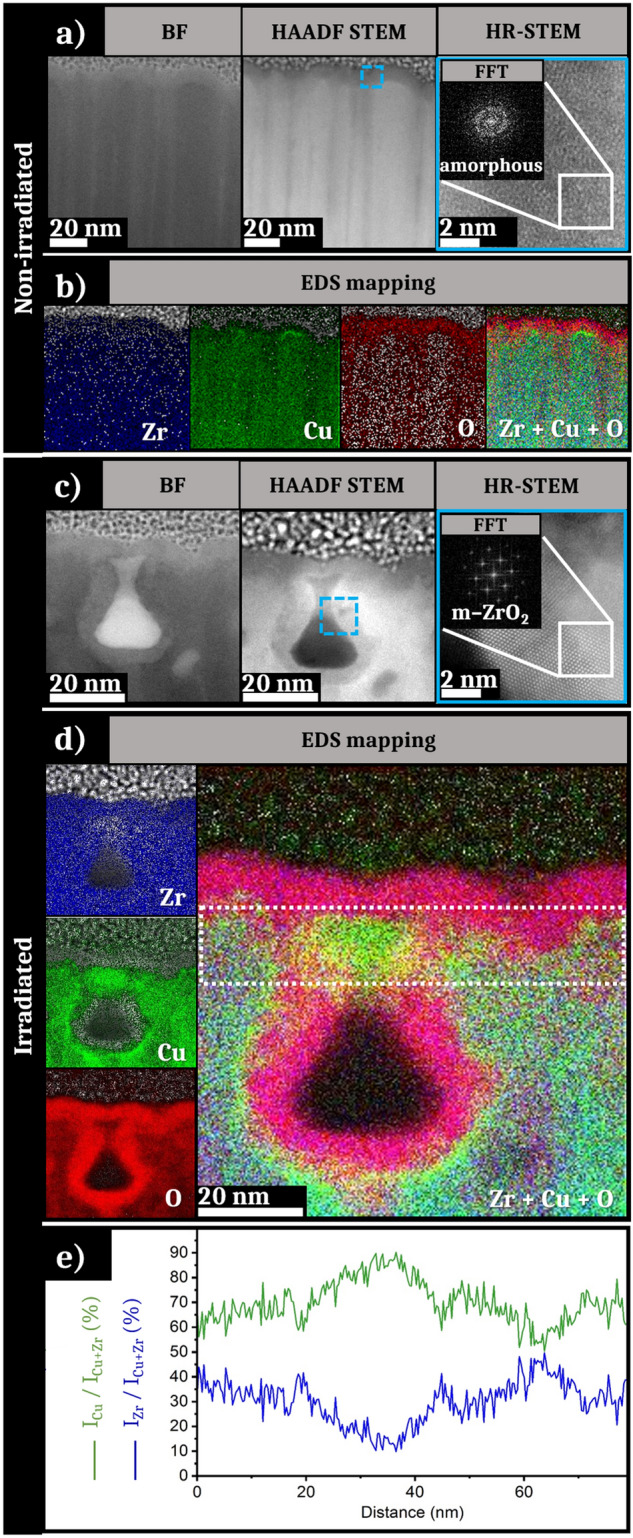
**a** STEM pictures in BF, HAADF STEM mode of a non-irradiated area, **a** HR-STEM image with the 2D-FT as inset reveals an amorphous structure. **b** EDS mapping of the STEM zone presented in (**a**). **c** STEM pictures in BF, HAADF STEM mode of a nanowell of a zone irradiated under the fluence of 0.06 J cm^−2^ with 50 bursts, **a** HR-STEM image on a location surrounded in blue with the 2D-FT as inset reveals a crystalline structure. **d** EDS mapping of the STEM zone presented in (**c**). **e** Evolution of the relative intensities of the *K*_α_ and *L*_α_ peaks of Zr and Cu in the white box area plotted in **d**

In addition to the STEM analysis, EDS mapping is performed on specific areas. In particular, locations showing changes in contrast and/or crystal structure after laser irradiation are more deeply investigated. Figure 4a presents the STEM images of the EDS mapped area on the non-irradiated sample in bright field (BF) and HAADF modes. A 2D-Fourier transform performed on the zoomed in picture confirms the absence of crystalline order. The EDS mapping of the selected area is exposed in Fig. 4b with the distribution of the different elements (zirconium, copper, oxygen) and finally the superposition of all of these elements. Zirconium appears to be homogeneously distributed in the entire sample in the FIB lamella, whereas such distribution is not observed for Cu and O. The first ten nanometers below the surface seem devoid of copper, while it is homogeneously distributed in the rest of the sample except under the ridges of some columnar structures where a stronger concentration scarcely occurs. In contrast, oxygen is sparse in-depth while a rich layer is observed on the surface. Regarding both the STEM pictures (Fig. 4a) and the EDS analysis with the superposition of all of the elements (Fig. 4b), the dark contrasted zones (surface of the sample and vertical columns) match with a zirconium-rich zone and a higher concentration of oxygen. Regarding the 2D-FT realized on the HR-STEM image in the contrast zone, this Zr and O-rich layer does not present atomic order. In the depth of the lamella, the amorphous structure is maintained. An extraction of the intensity of the *K*_*α*_ and *L*_*α*_ peaks of, respectively, Zr and Cu (not presented here) shows that the relative concentration of elements corresponding to the initial stoichiometry: ~ 65% of zirconium and ~ 35% of copper.

Figure 4c–e exposes the results for the ultrafast laser-irradiated lamella. EDS analysis is performed on two different zones characterized by the presence of an “open” and a “closed” NW. Both analyses giving similar results, we focus here on the analysis of the “open” NW. As previously, the zone of interest used for the EDS analysis of the irradiated sample with three STEM pictures in BF and HAADF STEM modes is presented in Fig. 4c. A 2D-Fourier transform of the HR-STEM image showing structural order is presented as an inset. Figure 4d shows the EDS mapping of the same area with four pictures showing the distribution of Zr, Cu, O and the superposition of all of these elements. Finally, Fig. 4e presents a graph of the relative intensity of the *K*_*α*_ and *L*_*α*_ peaks for the elements Zr and Cu, respectively. These are processed on the extracted zone shown by the white box in Fig. 4d. A layer of ~ 10 nm thickness exhibiting a high concentration of Zr and O is visible in Fig. 4d on the surface of the FIB lamella. This oxide thickness is similar to the one observed for the non-irradiated sample. The zirconium concentration appears relatively homogeneous in the sample except in the nanowell center. A lack of Cu is observed on the surface and in the zone surrounding the NW. Conversely, these copper-depleted areas are particularly rich in oxygen. This Zr and O-enriched layer matches with the dark-contrasted zone observable on the HAADF STEM image.

The HR-STEM image presented in Fig. 4c shows a crystalline structure surrounding the well with the presence of atomic planes whose structural order was confirmed by the Fourier transform. The analysis of the visible spots on the 2D-FT allows to determine the crystalline structure observed, that matches with monoclinic zirconia (*m*-ZrO_2_). Regarding Fig. 4e, the profile evolution of relatives *L*_*α*_ and *K*_*α*_ peaks for Cu and Zr is in accordance with the EDS mapping contrasts. The relative intensity of the peak of zirconium approach is almost stable all along the extracted zone. Nevertheless, a slight intensity increase is visible around the nanowell as well as on the surface of the sample. On the other hand, the intensity of the Cu peak is higher over the entire observed area. As shown by the green highlighting on the mapping, a slight increase in the copper peak intensity is observed around the layer of ZrO_2_. In these areas, the relative intensity reaches almost 90% compared to an average of 70%.

Single pulses experiments reveal experimentally the creation of nanowells regularly disseminated in the irradiated zone. In addition, nanocrystals of monoclinic ZrO_2_ are formed after laser irradiation. These results expose the opportunity to functionalize the Zr-Cu TFMG by two complementary ways: nanocrystallization and topographical modifications.

## Periodic Nanostructuring by Double-Pulse Laser Irradiation

For high numbers of laser shots, there appears a preferential orientation of structures parallel to the electric field polarization with a period near 100 nm. These structures are similar to HSFLs; however, they are hardly visible and cannot be distinguished from the original columnar morphology as seen on top surfaces of the STEM images.

**Fig. 5:**
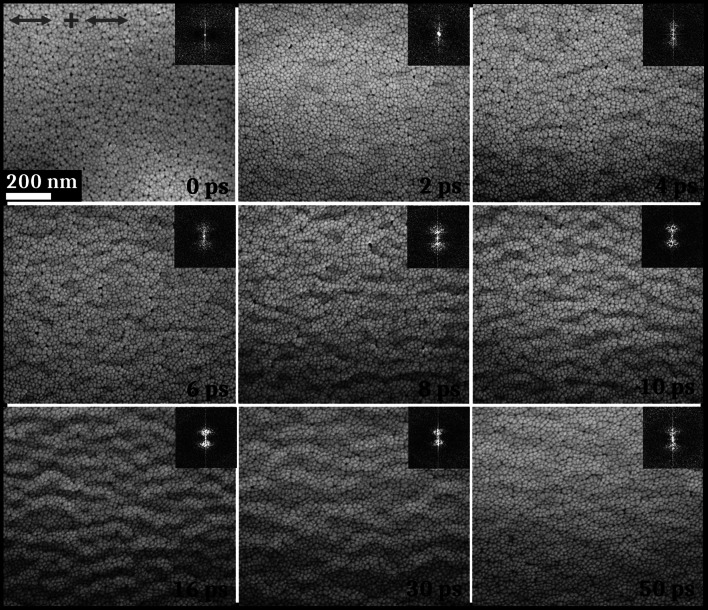
SEM pictures of the sample irradiated with 50 double laser pulses with horizontal collinear polarizations, with 0.06 J cm^−2^ of total energy; 2D-Fourier transforms as insets; the red arrows represent the ultrafast laser pulse polarizations

Controllable by adjusting the number of laser shots, the density of NWs stagnates after 20 laser pulses. As has been previously reported in the literature, temporal pulse shaping can improve the degree of control on the patterns at the nanoscale [18, 19]. Temporal laser beam splitting is thus performed with a double pulse setup with collinear polarizations (*cf.* Fig. 1c) in order to expand the nanostructuring possibilities. The effects generated by this temporal shaping on the characteristics of the nanowells (i.e., their size, density, and average distance between them) are observed. The energy condition is similar to the single pulses experiments with a total energy after combining the two pulses equals to 0.06 J cm^−2^. The number of laser shots is fixed at 50 and the delay between the two pulses changes from 0 to 70 ps. The results of these irradiations on the Zr-Cu TFMG are exposed in Fig. 5 with SEM pictures showing the evolution of the surface aspect of the irradiated area regarding the time delay between the two pulses. 2D-Fourier transforms are exposed as insets of each SEM picture.

The SEM picture corresponding to the laser irradiation with 0 ps of delay between both pulses shows a result similar to the irradiation with 50 single pulses. Moreover, according to the image analyses (not presented here), the created NWs have a density close to that found previously (near 650 NWs per µm^2^). With the evolution of the time delay, other structures (HSFLs) with a period close to 100 nm start to appear. Regarding the visual aspect of the SEM images and of the 2D-Fourier transforms as insets, the HSFLs and the associated Fourier spectrum contrasts increase between 2 and 16 ps where they reach an intensity maximum. After this delay condition, the contrast of the HSFLs starts to decrease until 50 ps. Finally, they are not observable for 70 ps of delay between the two pulses (not shown here). Parallel to the creation of HSFLs, NWs are still visible in the SEM pictures for each time delay condition. However, the concentration of nanowells decreases with the appearance of HSFLs. This concentration still reaches values of the order of a few hundred NWs per µm^2^, even if the density does not reach 600 µm^−2^. The average distance between the nanowells varies between 18 and 30 nm, in accordance with the average distances determined for single pulses experiments.

**Fig. 6:**
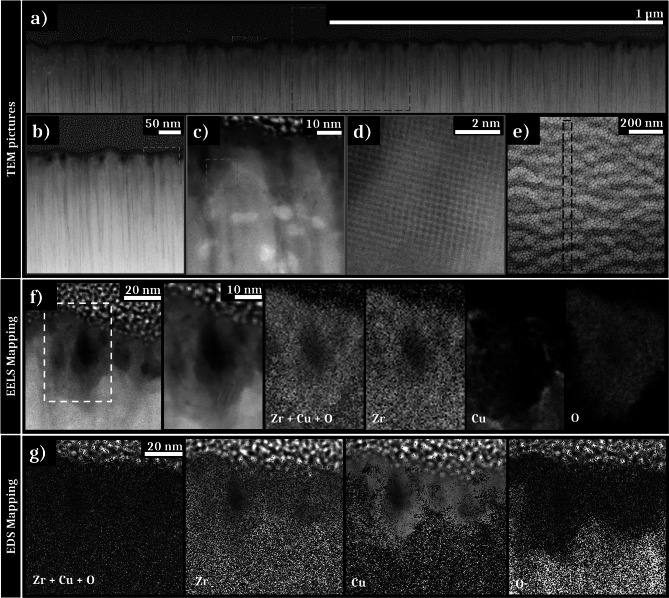
**a**–**d** HAADF STEM images of a FIB lamella extracted from a textured area irradiated under the fluence of 0.06 J cm^−2^ with 50 double pulses, showing HSFLs and nanowells on the sub-surface of the thin film. **d** shows columns of atoms characteristic of a crystalline structure surrounding a nanowell. **e** SEM picture of the FIB lamella extracted zone. **f** EELS mapping with, respectively, a STEM picture and a zoom of the analysis area, the overlap of the distribution of all of the elements, then the distribution of each element (Zr, Cu, and O). **g** EDS mapping with the overlap of the distribution of all of the elements, then the distribution of each element (Zr, Cu, and O)

As for single pulses, a FIB lamella is extracted from an area (Fig. 6e) irradiated with 50 double pulses with a total fluence of 0.06 J cm^−2^ and separated by 16 ps delay time. These conditions correspond to the maximal contrast of the created HSFLs. To reveal structural and chemical modifications, this FIB lamella is observed by STEM and spectroscopic analyses are performed, including EDS and EELS.

Figure 6a–d shows STEM images obtained in HAADF STEM mode with different zooms. The HSFLs depth is about 15 to 20 nm, explaining the low contrast observed by SEM analyses and the irregularity of the structures regarding the initial columnar profile of the TFMG. Nanowells are visible and randomly located all along the FIB lamella. Their morphology differs slightly from those obtained with the single pulses. The NWs appear to be smaller than in single pulses experiments and mostly “closed” below the sample surface, suggesting a more constrained cavitation process. A high contrast layer on the sample surface is visible along the entire FIB lamella. This also appears around the NWs as in the case of single pulses laser irradiations. With a thickness of about 10 nm, this layer contains nanocrystals as shown in Fig. 6d where atomic lattices oriented in different directions are visible. Regarding the complementary analyses EELS and EDS mapping (Fig. 6f and g, respectively), the dark contrasted layer in the surface and the zones surrounding the NWs contain mostly zirconium and a high oxygen concentration. Mappings show that these areas are extremely poor in copper. Fourier transforms performed on the crystallized areas reveal the creation of monoclinic zirconia after laser irradiation as observed for single pulses irradiation.

## Discussion

The nanocavities generated in metallic materials subjected to ultrafast laser irradiation are generally formed by cavitation mechanisms induced under a photomechanical regime [20–24, 48] The temperature gradients caused by the inhomogeneous laser energy deposition induce in-depth stress relaxation. This shock release leads to the formation of liquid layers that may be expelled by spallation and ultrafastly cooled and solidified [49]. All these mechanisms, reported both theoretically and experimentally, point out to the presence of solidified liquid bridges forming above the surface together with the emergence and coalescence of voids up to a few tens of nanometers below the surface [20, 21, 48, 49]. However, these nanocavities originate from local energy fluctuation and cannot be formed in a controlled and localized way in the material. They have random sizes and are primarily formed in mechanically weakened locations or locally enhanced optical coupling resulting from particular roughness before laser irradiation, for example polishing scratches [21].

Although free of large crystalline defects, TFMGs manufactured here present a singular columnar morphology due to the magnetron co-sputtering process. The width of their columnar growth is highly dependent on the composition of the film and can be tuned by the background argon pressure used during deposition [36]. The size of these columns as well as the roughness profile has a significant impact on the nanowells formation in the subsurface of the irradiated material [45].

In the present case, the interstices between the columns of the studied sample, visible in Fig. 1, may constitute privileged conditions for the local absorption of ultrafast light and constitute seeds of local field enhancement [22]. Then, the cavities form in the subsurface inside these interstices as shown in Fig. 3f. Remarkably, the nanowells are visible on the SEM images (Fig. 2a) as early as one laser shot. Up to two laser pulses, the width of the wells is larger compared to higher numbers of laser shots. The accumulation of laser pulses at the same impact point induces a negative feedback, and the “open” NWs progressively disappear. The TFMG morphology shows that the sample elaboration plays a key role in the homogeneous appearance of nanowells evenly distributed on the surface.

The regulated temporal energy feedthrough by double-pulse shaping subjects the material to high temperature as long as possible enabling to control its temperature-dependent viscosity [50] and cavitation phenomena. Periodic HSFL-like structures, visible in Fig. 5, appear due to capillary phenomena and their contrast increases with the time delay applied between the double pulses [45]. In parallel, NWs are also observed with modified aspect, concentration and size in comparison to those obtained in single pulses experiments.

Regarding the structural aspect, single and double pulses laser irradiations generate nanocrystals of monoclinic zirconia (*m*-ZrO_2_) on the film surface and around the NWs. Due to the initial stoichiometry of the film (Zr_65_Cu_35_) and the classical temperature ranges obtained with femtosecond laser irradiation, the metallic glass should pass in molten state (*T* > 1400 K) [36, 50]. Heating and cooling rates are highly elevated for ultrafast laser irradiation, promoting the stability of the amorphous structure. However, regarding chemical aspects, the air-interface interaction fosters zirconium oxide appearance before irradiation mainly due to the high affinity of zirconium for oxygen. A 10 nm native oxide layer is observed on the surface before irradiation. The generated monoclinic zirconia layer presents a comparable dimension. In terms of stability at room temperature, the monoclinic phase predominates [51]. This phase can be formed for pressure from 10^5^ to 10^10^ Pa. For temperatures between 2570 and 3000 K, the zirconia formed is in cubic form. Depending on the cooling and pressure profile, this cubic zirconia passes through a tetragonal phase and finally stabilizes in a monoclinic phase below 1400 K (at atmospheric pressure) [51]. This phase transformation may be associated with a small increase in volume of the unit cell coupled to a specific thermoplastic behavior [52].

In molten state, while zirconium atoms preferentially react with oxygen atoms to form ZrO_2_, copper atoms are still mobile in the mixture which will externalize them on cooling. Even if Cu and Zr melt present slightly different surface tension coefficients [53, 54], the thermophoresis effect, also known as Soret effect, may dominate atomic segregation for binary mixtures [55]. This phenomenon relies on thermodiffusion forces such as interface stresses and thermophoretic volume forces. While Zr atoms preferentially form ZrO_2_ crystallites, copper atoms agglomerate in liquid phase that solidifies in the hundreds of picoseconds timescale. These small agglomerates are visible around the nanowells on EDS (Fig. 4d) and EELS mappings (Fig. 6f).

**Fig. 7:**
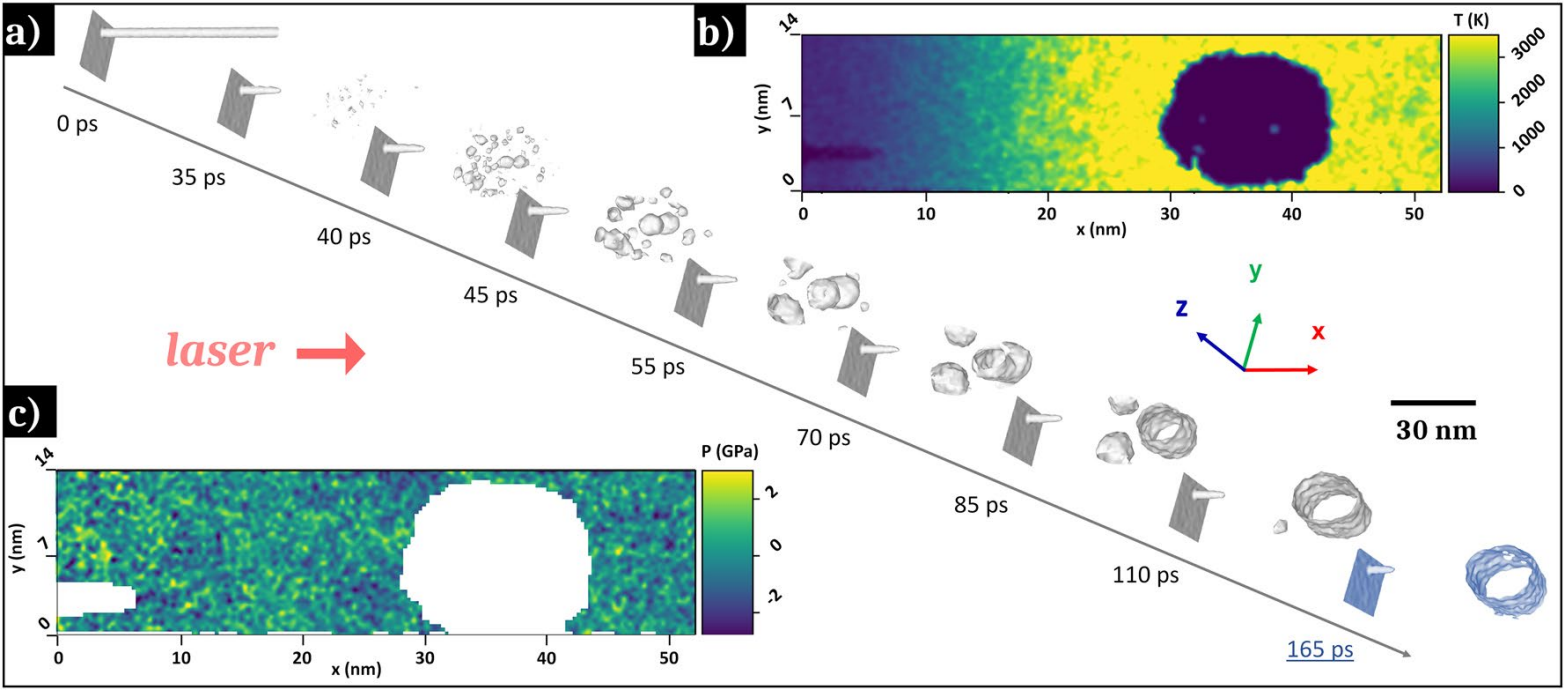
**a** Molecular dynamic simulation showing the development of nanocavities on a Zr-Cu metallic glass surface upon ultrashort laser irradiation with a peak fluence *F* = 0.06 J cm^−2^ and one laser shot. Evolution of **b** the temperature and **c** the pressure around a nanowell 165 ps after the start of the irradiation simulation

In order to confirm the mechanisms involved in the nanowells appearance preferentially inside the interstice and the creation of monoclinic zirconia, a laser irradiation simulation is conducted on a Zr-Cu metallic glass surface containing a pre-created interstice. Figure 7a shows the creation of nanocavities and their nucleation dynamics after laser irradiation. The evolution of the thermomechanical properties with the propagation of a temperature and pressure gradient mostly along x-axis and an expansion of the simulation box along this axis was observed (not visible in the figure). The simulation confirms the literature results showing the appearance of nanovoids and their coalescence [48, 49].

At the end of the simulation, a nanowell is obtained in the gap of the interstice present before irradiation. This well has a diameter of about 15 nm and appears 40 nm below the irradiated surface, which is similar to the characteristics of the nanowells obtained experimentally. Two maps of temperature (Fig. 7b) and pressure (Fig. 7c) around a nanowell are extracted. Regarding the temperature and pressure profiles shown in Fig. 7b–c, the pressures surrounding a typical nanowell during its creation are in the order of the GPa and the average temperature is around 3000 K. These results confirm that the metallic glass passes in a high-temperature molten state with reduced viscosity. Preferential oxidation of zirconium occurs as well as a diffusion of copper atoms before rapid solidification. During cooling process, zirconium nanocrystals stabilize in monoclinic phase, the most stable phase at atmospheric pressure and room temperature.

Zirconia, as a bulk material, has particularly interesting properties such as low thermal conductivity, high thermal expansion coefficient and ionic conductivity. Due to its high thermal expansion coefficient and heat stability, zirconia is frequently used as a thin film deposition on metals, serving as a thermal barrier and also modulating the surface properties of the coated material [56–58]. In addition to its biocompatibility, zirconium dioxide has optimal mechanical and fracture resistance properties [59]. As nanocrystals in a biocompatible metallic glass matrix, zirconia can further enhance the mechanical properties by, for instance, limiting crack propagation. By combining zirconia nanocrystals with a high concentration of nanowells on the surface, a wide range of applications can be considered, from renewable energy to biomedical applications with the storage of active species. The effective exposed surface actually increases by 65% from a 1 µm^2^ non-structured area to an irradiated one containing 650 nanowells. The surface irradiation by femtosecond laser pulses is thus a major asset in order to functionalize TFMG surfaces.

## Conclusions

The ultrafast laser irradiation of Zr_65_Cu_35_-based thin film metallic glasses experimentally and theoretically reveals the generation of nanowells regularly distributed on the surface of the film and able to initiate zirconia nanocrystals growth. These structural and topographical modifications occur simultaneously in a reproducible and controllable manner with single and double pulse laser irradiations. These results demonstrate the ability to control the entire elaboration process from the fabrication of a Zr-Cu thin film metallic glass with a singular columnar morphology that drives the generation of a double pattern composed of a controlled concentration and homogeneity of embedded nanowells and periodic nanostripes. These homogeneously distributed nanowells open the route to applications based on liquids storage at the nanoscale and/or the enhancement of the mechanical properties of the TFMGs.
